# iVR-fNIRS: studying brain functions in a fully immersive virtual environment

**DOI:** 10.1117/1.NPh.11.2.020601

**Published:** 2024-04-04

**Authors:** Ke Peng, Zahra Moussavi, Keerthana Deepti Karunakaran, David Borsook, Frédéric Lesage, Dang Khoa Nguyen

**Affiliations:** a University of Manitoba, Department of Electrical and Computer Engineering, Price Faculty of Engineering, Winnipeg, Manitoba, Canada; b Massachusetts General Hospital, Harvard Medical School, Department of Psychiatry, Boston, Massachusetts, United States; c Massachusetts General Hospital, Harvard Medical School, Department of Radiology, Boston, Massachusetts, United States; d University of Montreal, Institute of Biomedical Engineering, Department of Electrical Engineering, Ecole Polytechnique, Montreal, Quebec, Canada; e Montreal Heart Institute, Montreal, Quebec, Canada; f University of Montreal, Department of Neurosciences, Montreal, Quebec, Canada; g Research Center of the Hospital Center of the University of Montreal, Department of Neurology, Montreal, Quebec, Canada

**Keywords:** functional near infrared spectroscopy, virtual reality, immersive virtual reality, head-mounted display, cave, multisensory stimulation

## Abstract

Immersive virtual reality (iVR) employs head-mounted displays or cave-like environments to create a sensory-rich virtual experience that simulates the physical presence of a user in a digital space. The technology holds immense promise in neuroscience research and therapy. In particular, virtual reality (VR) technologies facilitate the development of diverse tasks and scenarios closely mirroring real-life situations to stimulate the brain within a controlled and secure setting. It also offers a cost-effective solution in providing a similar sense of interaction to users when conventional stimulation methods are limited or unfeasible. Although combining iVR with traditional brain imaging techniques may be difficult due to signal interference or instrumental issues, recent work has proposed the use of functional near infrared spectroscopy (fNIRS) in conjunction with iVR for versatile brain stimulation paradigms and flexible examination of brain responses. We present a comprehensive review of current research studies employing an iVR-fNIRS setup, covering device types, stimulation approaches, data analysis methods, and major scientific findings. The literature demonstrates a high potential for iVR-fNIRS to explore various types of cognitive, behavioral, and motor functions in a fully immersive VR (iVR) environment. Such studies should set a foundation for adaptive iVR programs for both training (e.g., in novel environments) and clinical therapeutics (e.g., pain, motor and sensory disorders and other psychiatric conditions).

## 1 Introduction

### 1.1 Background

**Fig. 1:**
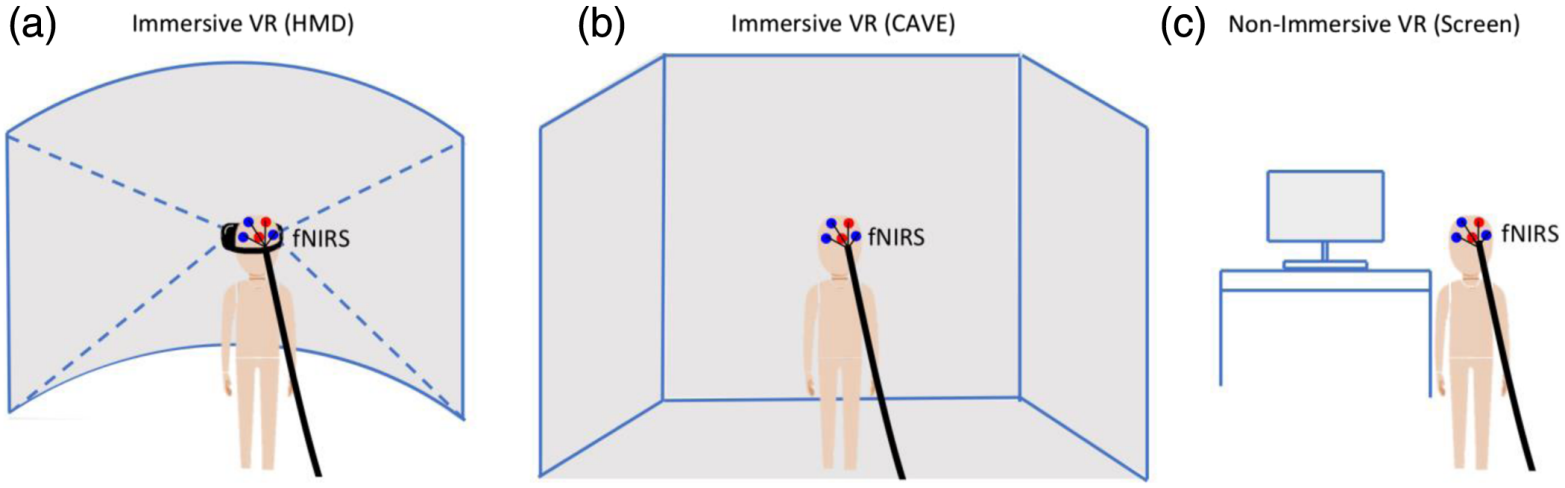
Depiction of immersive VR combined with fNIRS. (a) HMDs and (b) CAVE, compared with (c) non-immersive VR based on computer screens.

The concept of virtual reality (VR) can be tracked back to 1935 when American science fiction writer Stanley Weinbaum envisioned a device resembling goggles that could allow the wearer to experience “sight and sound, taste, smell and touch” and to interact with characters in a story. Today, modern VR technologies implement this concept by employing visual display units and projected environments to generate images, sounds, and other sensations that closely resemble reality to immerse a user in a virtual space.^1^ Fully immersive VR is most commonly achieved through the use of a head-mounted display (HMD), which contains small, high-resolution screens positioned in front of the user’s eyes enclosed with a goggle-like apparatus (Fig. 1). Other types of immersive VR utilize projections on screens installed on three or more surfaces surrounding the user within a cube-like room to enable an immersive feeling, a technique known as the cave automatic virtual environment (CAVE). By contrast, non-immersive VR experiences are generally realized through a conventional computer screen and an interface that would allow a user to observe or interact without altering the physical surroundings. For the purpose of this review, our focus is on studies that employ fully immersive VR (iVR) technologies.

The recent miniaturization of HMD-based iVR and the increased affordability of VR technologies have increased its popularity, extending its use beyond recreational purposes into scientific research and healthcare investigations.^2^ Although some early studies question the full reliability of iVR to mimic a real environment in terms of human performance,^3^ the advances in iVR have been successful at addressing those shortcomings by providing more realistic viewpoints and creating the sense of presence. In particular, iVR provides a useful tool in the study of brain functions and therapy, as perception, vision, and vestibular information to produce the feeling of presence and sense of immersion are constantly collected and analyzed by the user’s brain. Notably, iVR allows the researchers to deliver and precisely adjust multisensory stimulations to the brain in a safe and highly controlled environment that is often not feasible in real-world settings.^4^ Moreover, iVR may offer a cost-effective alternative to conventional stimulation methods to establish a similar realistic feeling, especially under the circumstances in which conventional methods are limited or unavailable (such as in underequipped hospitals/labs or at home).^5^^,^^6^ These applications encouraged methodological advancements that integrate iVR with neuroimaging techniques, enabling the delineation of users’ brain responses during their immersive virtual experiences.

### 1.2 Brain Measures

To date, electroencephalography (EEG) has predominately been integrated with iVR, typically through the installation of a VR HMD directly above the EEG cap.^7^ In cognitive and behavioral research, iVR-EEG has been employed to investigate various domains, including environment awareness, spatial navigation, attention, stress, emotion, and memory functions,^8^^–^^11^ by analyzing the evoked potential patterns and band power alterations associated with designed tasks in virtual environments. Another substantial body of iVR-EEG literature is focused on the development of neurofeedback systems and brain-computer interfaces, notably in training motor and executive functions for limb control, as seen in neurorehabilitation applications.^12^^,^^13^ In parallel, other groups have explored the feasibility of conducting functional magnetic resonance imaging (fMRI) scans with the user immersed in a virtual environment. Such studies generally applied MRI-compatible VR HMDs^14^ or utilized computer screens/mirrors placed at a close proximity to the user’s head inside an fMRI coil.^15^^,^^16^ Despite the progress, current iVR-EEG setups often necessitate compromises related to evoked potential signal complexity, reduced monitoring area, and susceptibility to electrical interferences.^17^^,^^18^ iVR-fMRI faces challenges from the high cost of the MRI console and MRI-compatible iVR devices, as well as the complicated implementation and synchronization requirements.^15^^,^^19^ Furthermore, EEG and fMRI are vulnerable to motion artifacts, which may be common in many VR applications involving large ranges of head or limb movement.^20^ Finally, fMRI scans restrict the user to a supine position within a noisy MRI room, potentially diminishing the level of immersion experienced by the user in the simulated virtual environment.^21^

Over the past decade, functional near-infrared spectroscopy (fNIRS) has attracted much attention in iVR studies.^22^^,^^23^ fNIRS is a noninvasive, flexible, and low-cost brain imaging technique that employs low energy near-infrared light to quantify cortical hemodynamic variations in terms of oxygenated hemoglobin (HbO) and deoxygenated hemoglobin (HbR) concentration changes. Therefore, fNIRS is generally less affected by electrical interference, making it highly compatible with the operation of HMD or CAVE equipment. Many available fNIRS devices feature high compactness and portability, simplifying the iVR-fNIRS setup for use in daily life scenarios (e.g., at home) or within complex clinical settings.^24^ The higher motion tolerance of fNIRS allows participants to undergo brain measures while maintaining a certain degree of mobility,^23^ which, in combination with its silence during operations, can significantly enhance users’ sense of immersion and extend the types of stimulations and tasks being administered.

In this paper, we review published work that integrated iVR and fNIRS in a concurrent setup and discuss the following topics: (1) the design and technical implementation of different iVR-fNIRS systems and studies; (2) major applications of iVR-fNIRS in neuroscience research and therapy; and (3) the advantages, current limitations, and future prospects of iVR-fNIRS. Based on the evidence, we provide an evaluation on the feasibility and usefulness of the combined iVR-fNIRS technique.

## 2 Literature Search

An English language literature search of VR and fNIRS was undertaken using the online public libraries PubMed^25^ and Web of Science^26^ on August 17, 2023. The following filtering terms were used to search paper titles and abstracts: “virtual reality” AND (“near-infrared spectroscopy” OR “NIRS” OR “diffuse optical tomography”). Additional strategies included manual searching for relevant publications from the selected papers’ reference lists, as well as utilization of PubMed’s “similar articles” function.

**Fig. 2:**
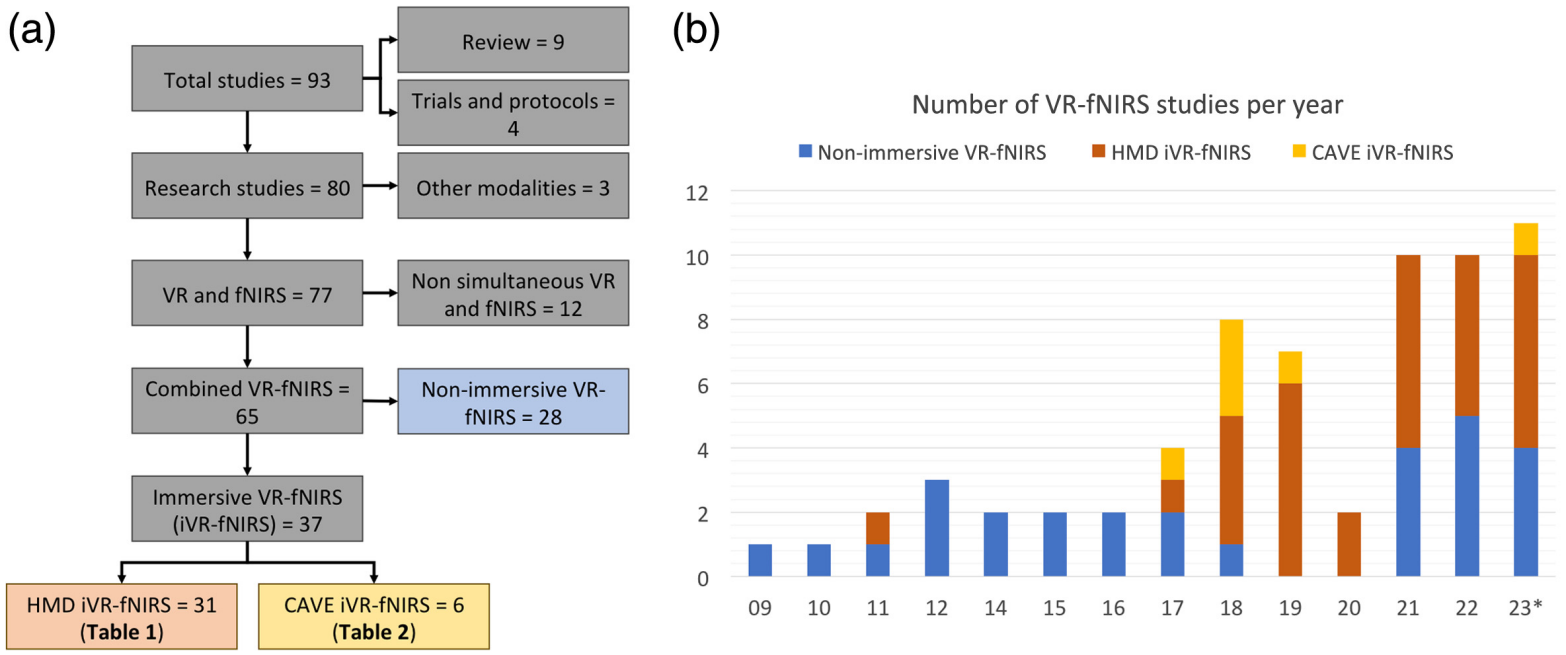
Published information. (a) Literature search results. (b) Number of identified VR-fNIRS studies by publication year. *Results based on literature search conducted on Aug 17, 2023.

The search returned a total of 92 papers after removing duplicates [Fig. 2(a)], including nine literature reviews (on various applications but not on the iVR-fNIRS technology), four published trials or research protocols, three studies using modalities other than fNIRS or VR, 12 studies in which fNIRS and VR were not used in a combined setup, 28 studies using non-immersive VR and fNIRS, and 37 studies employing iVR and fNIRS (31 studies using HMD and six studies using CAVE-like methods with an at least 180 deg field of view). Listing the papers by publication year [Fig. 2(b)], we noticed that the number of iVR-fNIRS studies underwent a significant increase since 2018 (34 out of 37, >91%), highlighting the increased popularity of using iVR and fNIRS to explore brain response in an immersive environment in recent years.

The summaries of the iVR and fNIRS system setup, analytical methods, and major findings of HMD VR-fNIRS and CAVE-like VR-fNIRS studies are reported in Tables 1 and 2, respectively.

**Table 1:** Summary of iVR-fNIRS studies employing HMDs.

	Subject (number)	VR	VR software	VR stimulation	Task design (Trial No.)	fNIRS device	Ch No.	Coverage	Measure (toolbox)	fNIRS findings
Seraglia et al.^27^	Healthy adults (8)	V8 Research HMD (modified)	3DStudio Max, Virtools	Interactive - cognitive control (Line bisection)	Block design (8)	ISS Imagent	20	Bilateral parietal and occipital lobes	HbO amplitude	HbO concentration increased in right parietal cortex during line alignment.
Hudak et al.^28^	Healthy adults with risk of ADHD (20)	Oculus Quest	KatanaSim	Interactive - attention and neurofeedback (Light control)	Block design (32)	Hitachi ETG-4000	8	Bilateral DLPFC	HbO amplitude (SPM8, Homer2)	VR training led to increased HbO concentration in following behavioral tests.
Dong et al.^29^	Healthy adults (10)	Oculus Rift	Unity, C#	Interactive - prospective memory (shopping)	Event-related (14)	Spectratech OEG-16	16	Bilateral frontopolar cortex	HbO amplitude	Subjects elicited higher HbO concentration levels in VR than in slide-based environments.
Gavgani et al.^30^	Healthy adults (9)	Oculus Rift	Commercial program (Archivision Helix)	Observational - cybersickness (rollercoaster ride)	Single task	Hitachi ETG-4000	52	Bilateral prefrontal, temporal and sensorimotor	HbO amplitude	Subjects experiencing sickness showed significant HbO increases in the parietotemporal regions
Landowska et al.^31^	Acrophobic adults (11)	Oculus Rift	Unity	Observational - fear of height (walking on a plank)	Single task	NIRx NIRSport	20	Bilateral PFC	HbO t-map (NIRSLab, SPM8)	Increased HbO activation was reported in the medial prefrontal cortex and DLPFC when exposed to virtual heights.
Lamb et al.^32^	Healthy young adults (25)	HTC Vive	NS/IH	Interactive - learning (interactive lecture)	Single task	Biopac fNIR devices Imager	16	Bilateral PFC	HbO amplitude (fNIR Software)	VR-based learning produced higher HbO changes in the PFC at a similar level to hands-on practice.
Hinderaker et al.^33^	Healthy young and old adults (21)	HTC Vive	Unity	Observational - posture control (optic flow)	Block design (5)	NIRx NIRSport	20	Left DLPFC and temporoparietal cortex	Hbo and HbR t-maps (NIRS Brain AnalyzIR)	Older adults showed higher HbO concentration in DLPFC than younger adults in balance control.
Jones and Ekkekakis^34^	Overweight adults (21)	Samsung Gear VR	Video	Observational - pleasure (music videos)	Single task	Artinis PortaLite	1	Right DLPFC	HbO and HbR amplitudes (Oxysoft, NIRS Analysis Package)	Low level of pleasure in fitness training was associated with higher HbO concentration.
Aksoy et al.^35^	Healthy adults (22)	HTC Vive	3DMedsim	Interactive - training (basic life support)	Block design (3)	Biopac fNIR	16	Bilateral PFC	HbO amplitude	Familiarization of task and training resulted in reduced HbO increases in the PFC.
Cheah et al.^36^	Healthy adults (11)	NS/IH	NS/IH	Interactive - behavior (food selection)	Single task	Artinis Octamon	8	Bilateral PFC	Hemoglobin amplitudes	The inferior and orbital PFC showed significant effect sizes on choices over high-density to low-density food.
Putze et al.^65^	Healthy adults (10)	HTC Vive	Unity	Interactive - memory (n-back)	Block design (10)	Artinis Oxymon	8	Bilateral PFC	HbO and HbR amplitudes	The authors were able to classify different VR workloads using HbO and HbR signal means.
Wang et al.^37^	Healthy adults (19)	HTC Vive	NS/IH	Interactive - wall breaking (creative tasks under embodied metaphors “breaking the rules”)	Single task	Hitachi ETG-7100	46	Bilateral PFC and right temporoparietal junction	HbO beta value (NIRS-SPM)	Increased creativity was associated with lower beta increments for HbO in medial PFC.
Shi et al.^38^	Healthy subjects (16)	HTC Vive	NS/IH	Interactive - learning and training (pipe operation under stress)	Single task	NIRx NIRSport	18	Bilateral DLPFC, motor cortex	HbO amplitude and functional connectivity	fNIRS showed significantly increased functional connectivity and HbO level from normal to stressful conditions.
Ge et al.^39^	Healthy young adults (20) and old adults (17)	Haofengyuan VRG	NS/IH	Interactive - gaming (avoid falling)	Block design (7)	Danyang Huichang Nirsmart	32	Bilateral PFC, MC	HbO beta values and functional connectivity (NIRSpark)	VR game elicited higher HbO beta values than mobile game in old adults. Brain connectivity was weaker in old adults compared with young adults.
Hu et al.^40^	Healthy adults (40)	Oculus Rift	NS/IH	Interactive - mindful breathing	Single task	TechEn CW6	45	Bilateral PFC, sensorimotor, visual	HbO t-maps and functional connectivity (NIRS Brain AnalyzIR)	Both traditional and VR breathing increased pain threshold.
Kuai et al.^41^	Healthy adults (24)	Oculus Rift	Unreal	Observational - behavior (speaking to public)	Single task	Hitachi ETG-7100	44	Bilateral temporal, frontal and parietal areas	HbO beta values (NIRS-SPM)	Speech performance was correlated with HbO beta values during speech delivery but not during anticipation.
Max et al.^42^	Healthy adults (30)	Oculus Rift	Unity, Blender-models	Interactive - behavior (food handling)	Block design (192)	Hitachi ETG-4000	24	Bilateral PFC	HbO amplitude	Interaction with food objects leads to enhanced HbO and HbR changes in the right DLPFC than office objects.
Tyagi et al.^43^	Healthy adults (34 fire fighters)	HTC Vive	NS/IH	Interactive - learning and retrieval (pipe operation under stress)	Block design (8)	NIRx NIRSport 2	21	Bilateral DLPFC, motor cortex	HbO amplitude and functional connectivity (Homer2)	To achieve similar retrieval performance, stress group presented increased activation in the motor area and frontal-motor connectivity.
Cho et al.^44^	Healthy adults (14)	Oculus Rift	Unity	Interactive - spatial awareness (right finger pointing)	Block design (50)	NIRx NIRScout	39	Bilateral frontoparietal network, motor cortex	HbO t-contrast (nirsLAB)	Right DLPFC and frontal eye field were activated during pointing task.
Deng et al.^45^	Healthy adults (15)	HTC Vive Cosmos	Commercial program (Beat Saber)	Interactive - gaming	Single task	Danyang Huichang NirSmart 6000A	44	Bilateral frontal, parietal and occipital areas	HbO beta value (NIRspark)	Increased immersion in VR was associated with better analgesic effect and higher HbO activations.
de With et al.^46^	Adults with fear of height (14) and controls (15)	Oculus Rift S	Unity	Observational - fear of height	Block design (3)	Artinis Brite 24	27	Bilateral PFC	HbO and HbR amplitudes (Artinis Oxysoft)	The right frontal HbO amplitude differences between ground and height conditions were greater in adults with fear of height.
Kaimal et al.^47^	Healthy adults (24)	Lenovo Explorer	Commercial program (Tilt Brush)	Interactive - creative self-expressive drawing	Block design (2)	Biopac fNIR Devices fNIRS sensor Imager 2000S	16	Anterior PFC	HbO amplitude (COBI Studio software)	Creative self-expression task resulted in lower PFC activity than the rote tracing, reducing PFC load.
Taguchi et al.^48^	Stroke patients (21)	Epson Moverio BT-300	Video	Observational - treadmill walking	Single task	Spectratech OEG-16	2	Bilateral PFC	HbO amplitude	Right PFC showed increased HbO during and after watching VR video of walking at a different speed.
Zapala et al.^49^	Healthy adults (12)	Oculus Quest	Unity	Interactive - attention and memory (2-back)	Block design (100)	Cortivision Photon Cap C20	10	Bilateral MFC and DLPFC	HbO and HbR beta values (OpenViBE)	HbO and HbR amplitudes in the DLPFC and MFG can be used to discriminate enhanced attention engagement and relaxation state.
Asaoka et al.^50^	Healthy adults (27) and kleptomania patients (11)	HTC Vive Pro	Video	Observational - behavior (store and outside sceneries)	Block design (3)	Hamamatsu NIRO-200	10	Bilateral PFC	HbO and HbR amplitudes	Kleptomania patients exhibited lower HbO increases and HbR decreases as well as weaker network strength within PFC in response to VR videos.
Betts et al.^51^	Healthy adults (10)	Oculus Rift	NS/IH	Interactive - spatial visualization (box arrangement)	Block design (60)	Biopac fNIR Devices fNIRS Imager 1200	16	Bilateral DLPFC and medial PFC	HbO and HbR amplitudes	Heavier task load and more difficult spatial characteristics led to higher HbO increases. Such increases could be attenuated by training.
Jones and Wheat^52^	Borderline overweight adults (12)	Oculus Rift	Unity	Observational - natural mountain forest cycling	Single task	Artinis PortaLite	1	Right DLPFC	HbO and HbR amplitudes	Less pleasant exercise was associated with greater prefrontal activations.
Kim et al.^53^	Healthy adults (24)	HTC Vive Cosmos Elite	Unity	Observational - motor (flying)	Single task	OBELAB NIRSIT	48	Bilateral DLPFC, FPC, VLPFC, and OFC	HbO amplitude	Increased accumulated HbO within the right OFC was positively correlated with user movement length and angle.
Pöhlmann et al.^54^	Healthy adults (30)	NS/IH	Commercial program (Helix rollercoaster)	Interactive – memory (n-back) during cybersickness (rollercoaster)	Block design (2)	Artinis Octamon	6	Bilateral DLPFC	HbO and HbR beta values and t-statistics (NIRS Brain AnalyzIR)	Right DLPFC activation was observed during memory tasks as well as during visual motion.
Tian et al.^55^	Old adults with mild cognitive impairment (17)	Oculus Quest 2	Commercial program (Beat Saber)	Interactive - motor (gaming)	Single task	Danyang Huichang NirSmart	18	Bilateral PFC, MC and occipital areas	HbO wavelet amplitude	The cognition level of older adults was correlated with PFC HbO amplitudes during interactive gaming.
Wiebe et al.^56^	ADHD patients (25 unmedicated+ 25 medicated) and healthy controls (25)	HTC Vive Pro	Unity, C#	Observational - attention and memory (working memory with distraction)	Block design (54)	NIRx NIRSport 2	10	Bilateral DLPFC	HbO amplitude (QT-fNIRS, homer3)	fNIRS showed no significant difference in DLPFC response to distractor among three subject groups.

Abbreviations. NS/IH, not specified or in-house developed; HbO, oxygenated hemoglobin; HbR, deoxygenated hemoglobin; PFC, prefrontal cortex; DLPFC, dorsolateral prefrontal cortex; MPC, medial prefrontal cortex; OFC, orbital prefrontal cortex; VLPFC, ventrolateral prefrontal cortex; ADHD, attention-deficit-hyperactivity-disorder.

**Table 2:** Summary of iVR-fNIRS studies employing CAVE-like environments.

	Subject (Number)	CAVE type	VR software	VR stimulation	Task design (Trial No.)	fNIRS	Ch No.	Coverage	fNIRS analysis	fNIRS findings
Unni et al.^57^	Healthy adults (19)	360 deg Projector, driving simulator	Virtual test drive	Interactive – cognitive control (driving) and working memory (n-back)	Block design (20)	NIRx NIRScout	78	Bilateral frontal, temporal and parieto-occipital areas	HbR amplitude (NIRSLab)	HbR levels in the bilateral inferior PFC and temporoparietal areas were able to predict working memory load during driving.
Hoppes et al.^58^	Healthy adults (15)	Three-screen projector	NS/IH	Observational - visual (optical flow)	Block design (10)	TechEn CW6	32	Bilateral frontotemporo-parietal and occipital lobes	HbO and HbR beta values	Viewing optic flow with a fixation target was associated with increased HbO and decreased HbR in fronto-temporal-parietal and occipital regions compared with stationary visual field.
Hoppes et al.^59^	Visual vertigo patients (15), healthy control (15)	Three-screen projector	NS/IH	Observational - visual (optical flow)	Block design (10)	TechEn CW6	32	Bilateral frontotemporo-parietal and occipital lobes	HbO and HbR beta values	Visual vertigo patients had decreased HbO in bilateral middle frontal regions when viewing optical flow on a fixed platform.
Landowska et al.^60^	Acrophobic adults (12)	Octagonal CAVE-like immersive projection	Unity	Observational - fear of height (walking on a plank)	Block design (3)	NIRx NIRSport	20	Bilateral PFC	HbO and HbR t-contrast (NIRS-SPM, NIRSLab)	Acrophobic patients activated the DLPFC and MPFC through learning of the fear stimuli, suggesting emotional inhibition to reduce fear response.
Scheunemann et al.^61^	Healthy adults (19)	360 deg Projector, driving simulator	Virtual test drive	Interactive - cognitive control (driving), visuospatial attention (lane width change), working memory (n-back)	Block design (2)	NIRx NIRScout	78	Bilateral frontal, temporal and parieto-occipital areas	HbR amplitude (NIRSLab)	fNIRS-measured HbR response associated with visuospatial attention and working memory showed significant interaction.
Stojan et al.^62^	Healthy older adults (49)	240 deg screen projector	D-Flow	Observational - motor (walking)	Block design (5)	NIRSx NIRSport	38	Bilateral frontal and parietal regions	HbO and HbR amplitudes (nirstorm)	Declines in performance from single task to dual task were accompanied by an upregulation in brain activation (higher HbO, lower HbR).

Abbreviations. NS/IH, not specified or in-house developed; HbO, oxygenated hemoglobin; HbR, deoxygenated hemoglobin; PFC, prefrontal cortex.

## 3 Current Design and Implementation of iVR-fNIRS Systems

### 3.1 iVR Implementation

**Fig. 3:**
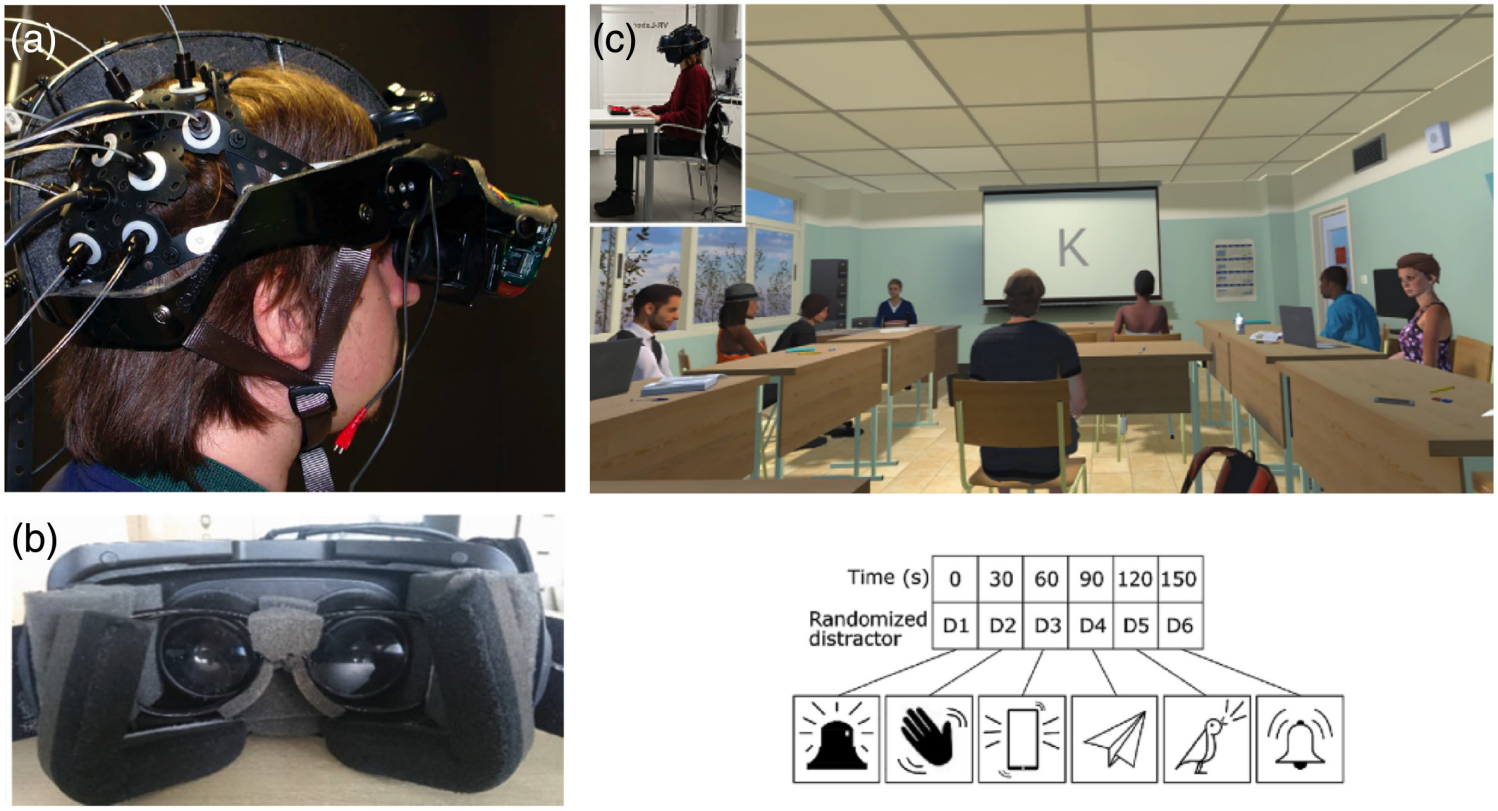
HMD iVR-fNIRS design in previous studies. (a) The first iVR-fNIRS combined setup by Seraglia et al.^27^ using a V8 Research HMD fixed on a modified bicycle helmet. Reprinted with permission under the CC-BY license. (b) Image of the HMD from Landowska et al.,^31^ in which modifications to the top part of the HMD have to be carried out to accommodate the installation of fNIRS optodes on the forehead. Reprinted with permission from Springer. (c) A virtual classroom environment with controlled distractions implemented by Wiebe et al.^56^ with HMD iVR to study adult attention deficit hyperactivity disorder. Reprinted with permission from John Wiley & Sons. Anyone wishing to use this figure will need to contact John Wiley & Sons publishing company directly.

**Fig. 4:**
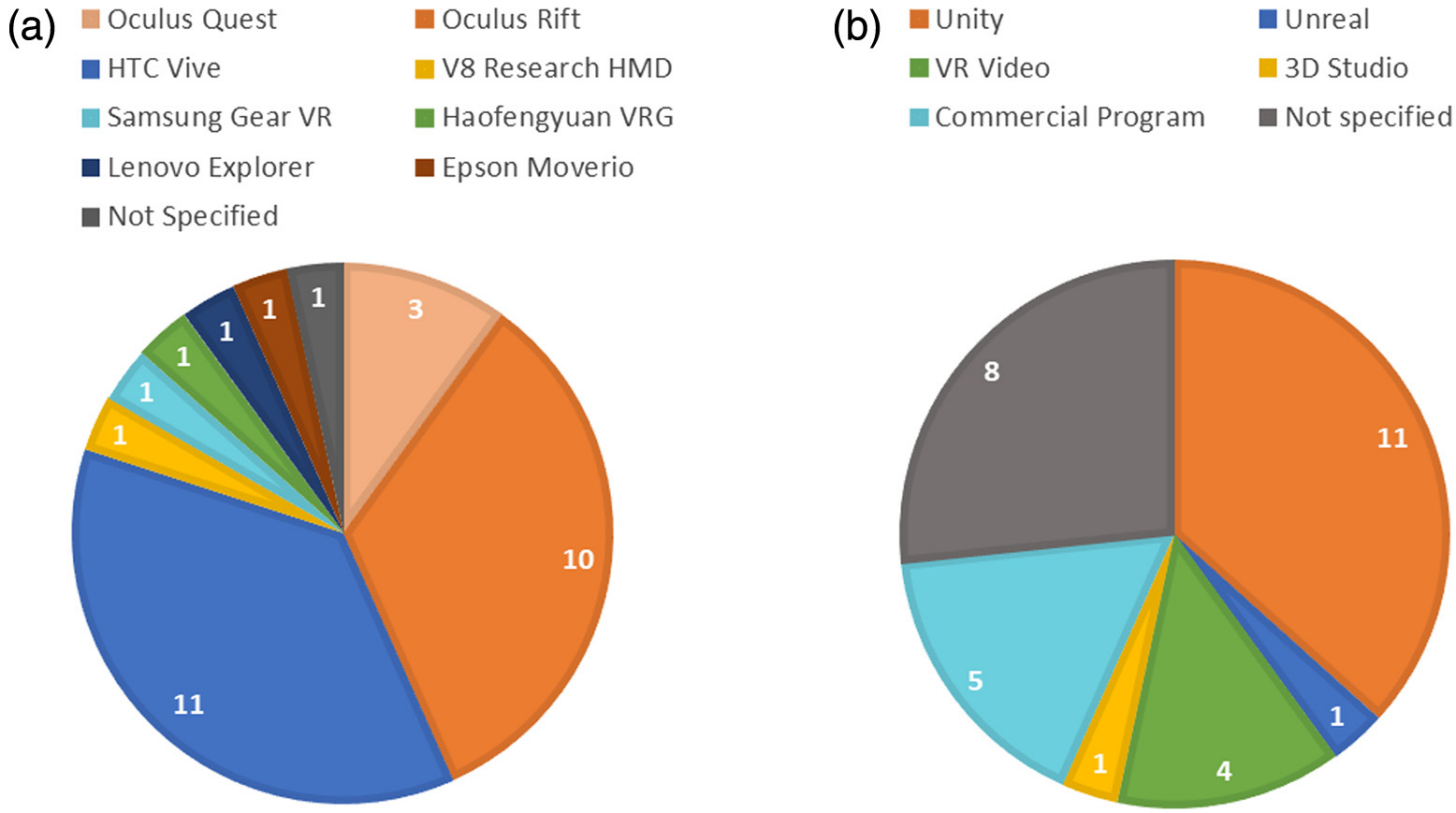
iVR products and use. (a) Brands and models of HMDs used in previous HMD iVR-fNIRS studies. (b) Methods for iVR task/stimulus development.

**Fig. 5:**
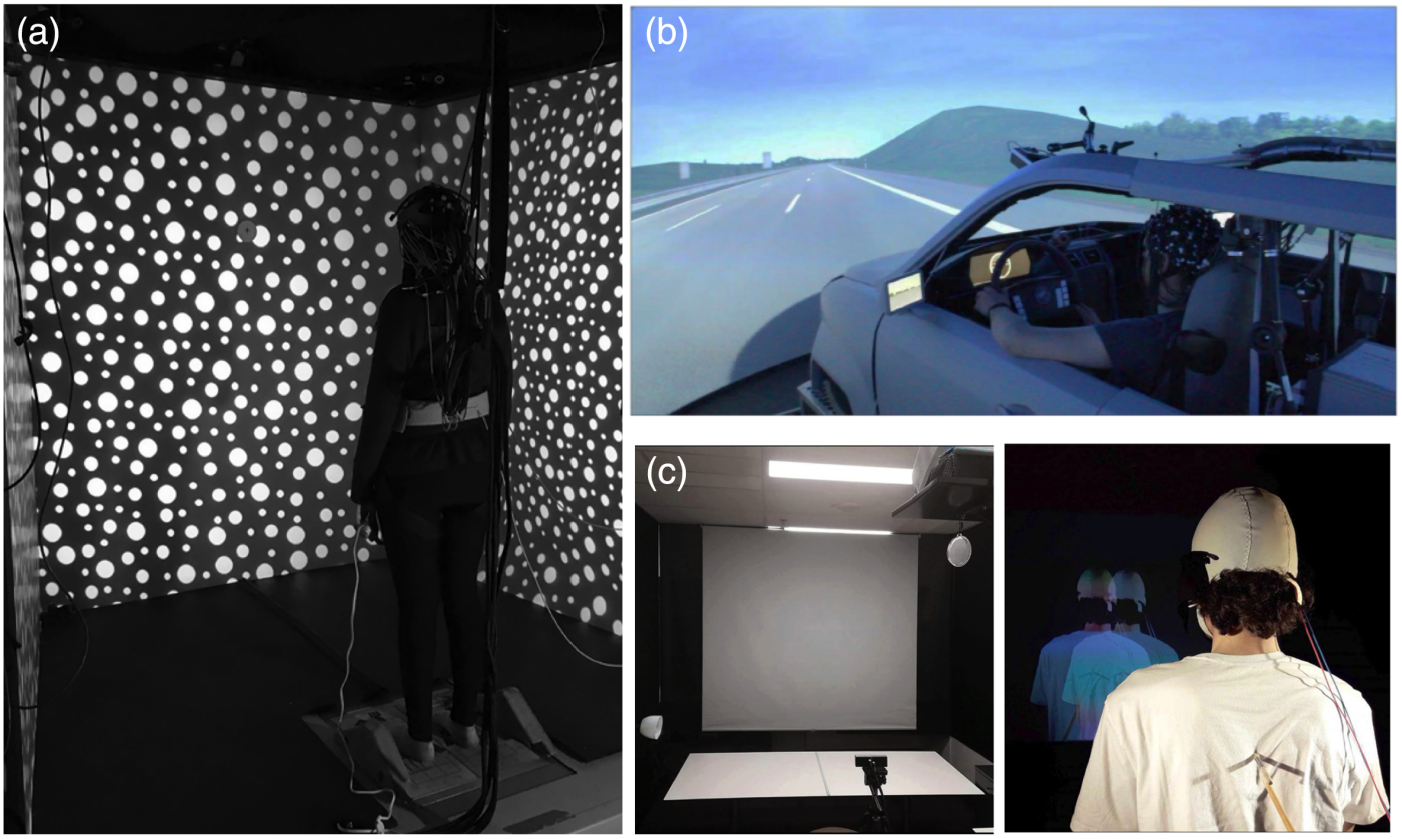
CAVE-like iVR-fNIRS design in previous studies. (a) Hoppes et al.^58^ utilized CAVE iVR and optical flow stimulations to explore brain responses in visual vertigo patients. Reprinted with permission under the CC-BY license. (b) A CAVE-like iVR-fNIRS setup was employed along with a full-size driving simulator to enhance subject immersion in the study of cognitive demands during driving.^57^ Reprinted with permission under the CC-BY license. (c) de Boer et al.^66^ presented a proof-of-concept design using CAVE-like iVR to create out-of-body experiences. Reprinted with permission under the CC-BY license.

The first attempt to install an iVR HMD along with fNIRS optode arrays was conducted by Seraglia et al.,^27^ who adapted a heavily modified V8 Research HMD fixed onto a bicycle helmet [Fig. 3(a)]. As VR HMDs have evolved to become more compact and affordable, recent studies predominantly employed commercially available HMDs. In some cases, modifications to the HMD or head strap have been necessary to accommodate fNIRS measurements, particularly in the prefrontal area [Fig. 3(b)]. Among the commercial VR HMD models, the most frequently used were the HTC Vive (HTC Corp., New Taipei, Taiwan), featured in 31% of the studies, and the Oculus Rift (Meta Platform Technologies, Menlo Park), employed in 28% of previously published work (Fig. 4). Standalone iVR HMDs (also known as all-in-one HMDs) such as the Oculus Quest, which incorporate built-in processors without any wired connection to a control computer, may be more favorable in studies that prefer a fully wireless and portable iVR-fNIRS setup^49^ or involve subject movement.^55^ CAVE-like iVR has been generally established with multi-screen displays or wide-angle projectors (Fig. 5). Compared with HMD, CAVE-like iVR-fNIRS studies are less common, likely due to the relatively higher costs and spatial demand. However, CAVE-like environment could offer distinct advantages, such as the ability to accommodate additional equipment or components (e.g., a full-size driving simulator) to further enhance user immersion.^57^^,^^61^ More discussions about HMD versus CAVE are provided in Sec. 5.2.

The induced iVR stimuli can be either “passive” (or “observational”) or “interactive.” Passive iVR involves immersing users in a virtual environment through HMD or CAVE, but their activities are limited to mainly observation and exploration. In previous iVR-fNIRS studies, passive stimuli were often delivered through the playback of pre-recorded three-dimensional (3D) videos or display of pre-configured virtual scenes. They were more prevalent in studies with a primary goal of providing an observational experience of distinct virtual environments. Conversely, interactive iVR tasks involved a higher degree of user interaction within VR, including object manipulation, action execution, and the ability to control/influence the course of events using additional hardware such as VR controllers. Those tasks were often employed in studying user behavior (e.g., in neuropsychological studies), as well as cognitive control. Most iVR-fNIRS studies that utilized in-house developed iVR tasks or stimuli opted for the open-source Unity 3D engine (Unity Technologies, San Francisco) and used C# programming language^63^ [Fig. 4(b)]. This is potentially because of its cross-platform compatibility with various types of VR headsets, including those manufactured by Oculus and HTC; its user-friendly development interface that includes pre-packaged virtual object assets and templates; and the abundance of supportive resources available to developers.^64^

In 59% of the reviewed studies, multiple iVR stimuli were delivered using a block design within one data acquisition session at a relatively constant time interval or through several sessions. Notably, 38% of the previous work employed a single continuous stimulation task, primarily to simulate real-life scenarios without habituating participants to the created virtual environment (e.g., in the study of phobia,^31^^,^^41^ pleasure,^34^ or creativity^37^).

### 3.2 fNIRS Implementation

Previous iVR-fNIRS setups incorporated a wide range of fNIRS devices with regards to brands and models, optode types, and montages, demonstrating the adaptability of different fNIRS systems in such study designs. Most of the work utilized a continuous wave fNIRS system, which maintains steady illumination of brain tissue and detection of transmitted near-infrared light intensities, and yielded relative changes of HbO and HbR concentrations through the differential pathlength approach. One study employed a frequency domain fNIRS system (specifically the ISS Imagent from ISS Inc., Champaign).^27^ However, the study only analyzed direct current component of the optical signals, resulting in relative hemoglobin concentration outputs.

**Fig. 6:**
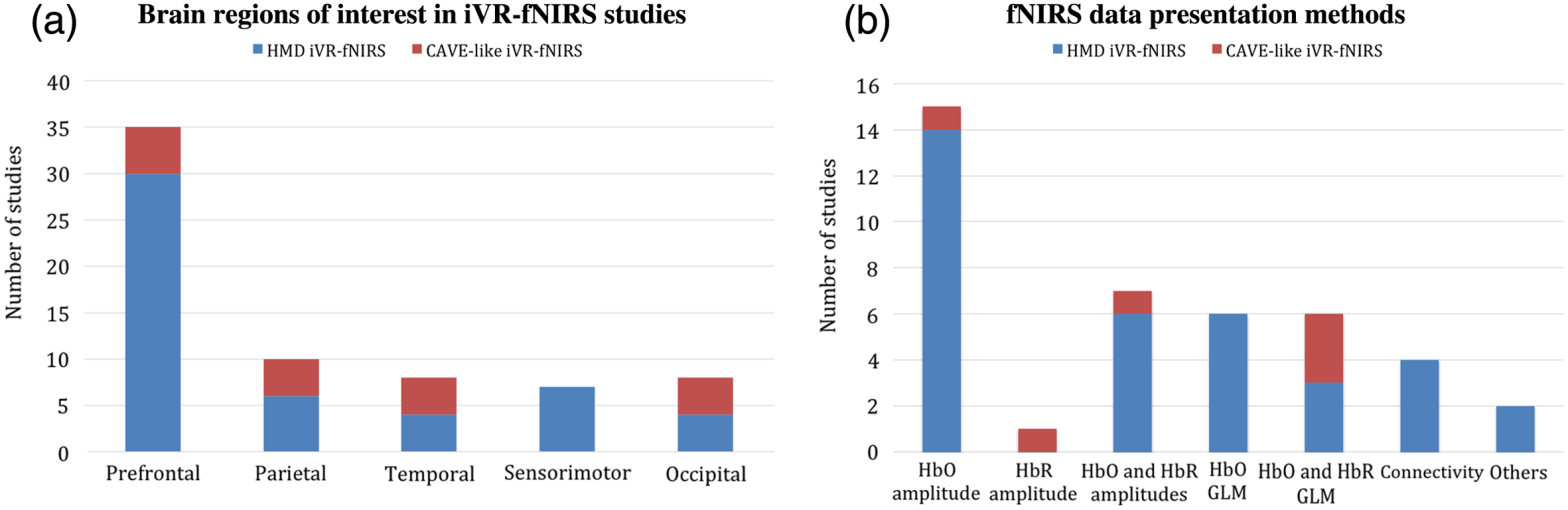
fNIRS measures in iVR-fNIRS studies. (a) Depiction of the major brain regions of interests covered by fNIRS. (b) Data presentation methods in previous iVR-fNIRS studies.

A smaller number of fNIRS channels were installed in iVR-fNIRS studies using HMDs (mean = 22, ranging from 1 to 52) compared with those with CAVE-like iVR environments (mean = 46, ranging from 20 to 78). This may potentially be due to the competition of surface space on subject head between fNIRS optodes and iVR HMDs. Targeted brain regions included primarily the prefrontal cortex (in 34 studies), most often the dorsolateral prefrontal cortex (DLPFC) and the frontopolar cortex (FPC) [Fig. 6(a)]. Other brain regions of interest that were frequently involved were the temporoparietal areas (10 studies), the sensorimotor cortex (seven studies), and the visual cortex in the occipital lobe (eight studies).

The analysis and interpretation of data in previous VR-fNIRS work largely relied on direct measures of relative changes in HbO and/or HbR concentration amplitudes [Fig. 6(b)]. Comparisons were conducted on either hemoglobin response peak values, areas under curve, or beta values and t statistic values extracted from a general linear model (GLM) analysis assuming a canonical shape of brain hemodynamic response. In addition, four studies explored the changes in functional connectivity of remote brain regions by computing the Pearson’s correlation coefficients between fNIRS signal time courses of different channels.^38^^–^^40^^,^^43^

## 4 Applications of iVR-fNIRS in Neuroscience Research and Therapy

### 4.1 iVR-fNIRS in Cognitive Neuroscience

iVR-fNIRS has been employed in cognitive neuroscience research, offering insights into the neural correlates of various processes including cognitive control,^27^^,^^39^ prospective memory,^29^ working memory,^65^ and attention.^49^ iVR can create controlled testing environments that isolate the subjects from external interferences; therefore it was particularly useful in studies that required high levels of subject attention and engagement. For example, Zapała et al.^49^ designed attention and working memory tasks in iVR and reported a higher accuracy in distinguishing users’ attention state and resting state with fNIRS-measured PFC signals compared with previous studies that did not utilize iVR. Conversely, iVR-fNIRS could facilitate the study of attention and cognition-related deficits through controlled distractions. The recent work of Wiebe et al.^56^ sought to assess adult attention-deficit hyperactivity disorder (ADHD) with combined iVR, EEG, and fNIRS by immersing their patients in a virtual classroom with induced visual, auditory, or audiovisual distractions [Fig. 3(c)]. A proof-of-concept neurofeedback system was designed in another study^28^ to train adults who were highly impulsive for ADHD using cognitive control tasks in a simulated virtual classroom. Their results showed a significant reduction in subject impulsive behaviors during follow-up tests as well as improved abilities to regulate PFC activities.

Several iVR-fNIRS studies have investigated brain-level interactions related to cognitive loads in dual or multiple task experiments, leveraging the ability of iVR to precisely deliver multisensory stimulations in a controlled environment. One such application explored the brain resource demands associated with driving^57^^,^^61^ [Fig. 5(b)]. Using a CAVE-like iVR setup and a driving simulator, healthy participants were requested to engage in multiple subtasks involving visuospatial attention (lane width change) and working memory (vehicle speed adjustment) functions during realistic highway driving scenarios.^61^ Simultaneous fNIRS measurements revealed that brain activity changes in the DLPFC and the parietal lobe were dependent on both participants’ visuospatial attention levels and working memory loads in high-demand driving situations, suggesting significant interactions among the underlying neural processes and competition for brain resources. In a separate study, Stojan et al.^62^ investigated brain activation changes from single-task to dual-task walking in older adults with working memory and inhibitory control tasks. They reported increased activations in the ventrolateral prefrontal area and parietal lobe accompanied by deteriorated task performance, indicating neural inefficiency in older adults under heavy cognitive loads.

iVR perhaps offers an unparalleled platform for researchers to manipulate a user’s perception of space and time. In Cho et al.,^44^ the authors described their virtual prism adaption platform integrated with iVR and fNIRS for correcting unilateral spatial neglect in stroke patients. In this setup, a virtual hand was created and was intentionally misaligned with the user’s actual hand. The virtual hand was then used to direct the user’s hand to point at targets placed at the neglected side of space. Validations in healthy subjects revealed significant activations in the DLPFC and the frontal eye fields, both components of the dorsal attentional network. Other intriguing applications included the use of VR and live streaming images to induce visual illusions for studying out-of-body experiences^66^ [Fig. 5(c)]. These discussions highlighted the potential of combining iVR and imaging techniques in delineating brain functions in rare and unpredictable scenarios that may be challenging to replicate in real-world environments.

### 4.2 iVR-fNIRS in Behavioral Research

iVR-fNIRS finds applications in the study of various human behaviors, such as decision-making,^36^^,^^42^ creativity,^37^ and self-expression.^47^ In an iVR-simulated food selection scenario, Cheah et al.^36^ explored the role of the inferior and orbital PFC in regulating user’s choices between high-nutrition-density and low-density-nutrition foods. Two recent studies investigated how emotion may influence decision-making regarding exercise and physical activities.^34^^,^^52^ Their findings showed that individuals exposed to more pleasant exercising environments exhibited weaker brain activations in the DLPFC, reflecting less cognitive effort in retaining a positive affect and exercise interest. In the study of creative behaviors, Wang et al.^37^ placed a virtual wall in iVR to obstruct a corridor, and discovered that subjects encouraged to break the wall performed better in subsequent creativity-demanding tasks and exhibited a lower level of brain activations in the medial PFC during the tasks. Similarly, Kaimal et al.^47^ reported reduced activations in the anterior PFC during creative self-expression drawing in iVR compared with rote tracing. These results implied that the frontopolar area might potentially be involved in rule-based and self-restrictive behaviors.

iVR-fNIRS was also used to unveil the functional aspects associated with learning of knowledge/skills using new VR-based teaching tools in education. Lamb et al.^32^ compared brain activity levels across different types of teaching methods in a simulated biology class and observed higher HbO changes in the DLPFC and better learning outcome during iVR-assisted interactive practice than the more conventional video lectures. Another study on the training of spatial visualization abilities in iVR reported similar activations in the DLPFC and orbitofrontal areas, which were positively correlated with task difficulty and modulated after practice and familiarization of the task.^51^ These results demonstrated the role of DLPFC in critical thinking, memory, and motor control.^67^ Several studies have employed iVR-fNIRS to assess user performance in job-related skill learning, such as in basic life support training,^35^ industrial shutdown maintenance,^38^ and firefighter pipe operations.^43^ Those investigations reported a consistent increase in brain activities in the DLPFC during skill acquisition and retrieval, which were enhanced under induced stress (e.g., adding a time limit) and attenuated after repetitive training.^35^

iVR has been applied in treating specific phobias and anxiety disorders by setting up virtual environments that expose the patients to their feared objects or environments in the absence of actual harm. Using fNIRS to simultaneously record the brain responses, previous iVR-fNIRS work has explored the neural correlates of acrophobia (i.e., fear of height)^31^^,^^46^^,^^60^ and public speaking anxiety.^41^ In both cases, exposure to feared situations led to greater activations in the DLPFC and the medial PFC, which might reflect modulated emotional processing in the PFC and subcortical areas. With a similar idea, a study employed iVR environments to trigger addicted behaviors.^50^ Their work on individuals with kleptomania revealed distinct PFC activation and connectivity patterns in response to 3D videos of shops/markets compared with healthy controls.

### 4.3 iVR-fNIRS in Postural Control and Locomotor Abilities

iVR-fNIRS offers a versatile platform to assess balance and related motor functions with either a treadmill^48^ or the use of optical flow^33^^,^^58^ [Fig. 5(a)]. Optical flow induces an illusion of movement to a stationary observer by moving objects in a virtual scene relative to the observer.^68^ With a CAVE iVR setup, Hoppes et al.^58^ compared fNIRS-measured brain signals in healthy subjects during exposure to optical flow versus an unchanged visual field. They observed higher brain activation levels in the fronto-temporo-parietal area and the occipital lobe when the subjects viewed optical flow on a fixed surface. These changes were presumably associated with related vestibular activities for postural stabilization. Extending their study to patients with visual vertigo, the same research group reported similar brain activations in the temporal and occipital regions but deactivations in the middle prefrontal area in visual vertigo patients compared with healthy controls, which they attributed to vestibular hypofunction.^59^ Interestingly, Hinderaker et al.^33^ employed optical flow stimulations through a iVR HMD and discovered reduced brain activations in the fronto-temporo-parietal areas and the frontal cortex in older adults compared with young adults, particularly under fast optical flow speeds. These brain changes might be associated with reduced ability to process visuosensory information and to maintain postural equilibrium in visual vertigo patients and older adults.

Cybersickness, a specific form of motion sickness triggered solely by visual stimuli (i.e., illusory of self-motion),^69^ has been the subject of two investigations with iVR-fNIRS. In Gavgani et al.,^30^ the authors immersed healthy volunteers in a virtual rollercoaster ride and observed elevated HbO concentration levels in bilateral temporo-parietal regions among participants who experienced strong motion sickness symptoms. Pöhlmann et al.^54^ conducted a similar experiment involving a virtual rollercoaster scenario but introduced simultaneous working memory tasks within the iVR environment. They observed activations in the right DLPFC of their participants during both the memory tasks and periods when cybersickness was experienced. They proposed that user engagement in cognitive tasks might distract iVR users from cybersickness symptoms, potentially through a competition of attentional resources in the brain. Motion sickness and cybersickess are important issues for iVR.^70^ In Sec. 5.3, we provide further discussions on their implications in iVR-fNIRS research.

Additional studies have explored the perceptual and executive processes associated with sensorimotor functions in various iVR scenarios. For example, Kim et al.^53^ simulated an open sky space that allowed their participants to “fly freely” with few restrictions on the extents and angles of limb movements. They demonstrated a positive correlation between ranges of participant movements in iVR and the HbO concentrations in their right orbitofrontal cortex. Tian et al.^55^ utilized fNIRS to assess brain conditions in older adults with mild cognitive impairments while they engaged in an iVR video game requiring large extents of upper limb movement. They observed significant lower brain activation levels in the prefrontal and occipital areas in the patient group with lower Montreal cognitive assessment scores, reflecting potentially more impaired motor control abilities.

### 4.4 iVR-fNIRS in Pain Management

It has been reported that VR might modulate users’ perception of pain by providing effective distractions and reducing the unpleasantness/distress associated with pain experiences.^71^^,^^72^ Two iVR-fNIRS studies aimed at delineating the underlying neuronal processes in pain reduction. In Deng et al.,^45^ an immersive video game was employed to divert the attention of study participants while electrical pain stimuli were applied to their back. The results showed significant brain activations particularly in the DLPFC and the premotor cortex. Both regions were suggested to be involved in attention orientation and top-down antinociceptive control.^73^ Hu et al.^40^ used iVR to explore the brain mechanism of mindful breathing in the modulation of induced thermal pain at the trigeminal nerve. Their findings revealed that meditation raised subject pain thresholds potentially through the enhancement of the brain functional connectivity particularly within the anterior PFC, as well as between the PFC, premotor cortex, and auditory/visual regions. These brain areas are believed to play important roles in regulating attention and high-level integration of multisensory information.^74^

## 5 Discussion

### 5.1 Use of iVR in fNIRS Research

The most prominent advantage of using iVR to deliver stimulations/tasks to evaluate associated brain responses is its ability to improve ecological validity in method assessment.^75^ Fully immersive VR setups, such as HMDs or CAVE environments, are capable of providing complex, three-dimensional and realistic testing conditions that are highly comparable to users’ daily environments. Traditional tests on cognitive functions and behaviors have often been criticized for their lack of ecological validity, which can lead to discrepancies between test results and real-life performances.^76^ For example, several assessments of ADHD in children revealed that laboratory assessments of inattention, impulsivity, and overactivity showed only low-to-moderate consistency with measures conducted in more natural at-school or at-home settings.^77^ By contrast, ecological approaches (e.g., conducting a study during lectures given in a real classroom) lack quantitative/normative data, offer less specific assessments, and have a low reliability due to many factors that cannot be controlled.^78^ The recent development of iVR-fNIRS methods, on the other hand, allowed the researchers to bring ADHD patients to a virtual classroom, assessing their attention and memory functions during virtual lectures with precisely controlled distractors.^56^ The use of iVR significantly enhances the verisimilitude and veridicality of the study by providing measurements of brain functions that are representative in users’ normal living conditions and predictive of their daily behaviors outside the test environment,^79^ while maintaining scientific rigor and reproducibility.

iVR can create simulated testing environments or deliver stimulations that may be restricted in real world situations or “impossible” based on the physics laws of nature.^80^ This flexibility allows researchers to explore brain activities during various neurological and neuropsychological processes that were previously deemed difficult or risky in a laboratory setting.^81^ Several iVR-fNIRS studies have implemented fully immersive virtual environments and stimulations that could span over multiple dimensions to modify subjects’ visual, auditory, haptic, and other sensory inputs, inducing illusions of flying, driving, out-of-body experiences, or emergency situations.^43^^,^^53^^,^^61^^,^^66^ Another application is the study of brain responses during exposure therapy for individuals with phobia or anxiety disorders.^60^ iVR allowed for complex object/situation presentations while offering precise adjustments on exposure type, duration, and dose, overcoming the current barrier of ethical or tolerability concerns in such studies.^82^ Although less explored in the current iVR-fNIRS literature, iVR has the potential to facilitate brain research involving patients with reduced mobility or cognitive impairment by providing tailored virtual environments and stimulations that accommodate specific test requirements, offer new experiences, or be used as alternatives to existing stimulation modalities.^5^ This may hold particular promise for neurorehabilitation, which aims to use repetitive training to promote neural activations in neurology patients to restore motor and executive functions after brain disorders or trauma.^83^ Indeed, physical therapy combined with iVR exhibits greater improvements in gait and balance than traditional rehabilitation approaches.^84^^,^^85^ The combination of iVR and brain imaging techniques may lead to further development of patient-specific approaches as a standalone or complementary tool for evaluating and rehabilitating brain functions in these populations.^86^

Finally, iVR offers a compelling advantage in enabling researchers to control the timing and intensity of multiple sensory stimulations while isolating research subjects from unwanted interferences in an immersive and enclosed environment. This provides a robust platform to study the integration and interaction of brain processes associated with simultaneously delivered stimulations or tasks.^87^^,^^88^ Past iVR-fNIRS studies have focused on assessing the interaction of cognitive control, visuospatial attention, working memory, and motor functions in scenarios such as distracted driving^57^^,^^61^ and multitask walking^62^ to explore the resource demand and brain performance during demanding tasks.

### 5.2 HMD iVR Versus CAVE iVR to Combine with fNIRS

HMD and CAVE represent two distinct approaches to establish iVR.^89^ When compared with CAVE, HMD excels in terms of cost, ease of system setup, and the ability to create a personalized virtual experience with the environment responding to the user’s head movement. HMD iVR headsets are generally affordable, usually ranging from under one thousand US dollars to a few thousands, whereas a complete CAVE setup can cost ten or a hundred times more depending on factors such as size, projection surface, and intended use.^90^ HMD iVR systems do not require large, dedicated study spaces or the complex installation of projectors/screens, making it ideal for applications that prefer a fully portable and flexible iVR-fNIRS setup. Additionally, HMD iVR may induce less ambient light interference with fNIRS signals, as the display screens are normally fully enclosed within the VR goggle.^31^ By contrast, CAVE iVR implements motion tracking cameras to track body movements or as a means of interaction with the virtual space.^91^^,^^92^ Such cameras (e.g., time-of-flight depth cameras^93^) often use infrared light, which represents a source of interference for fNIRS. It is worth noting that caution should also be exercised in the case of HMD iVR with the eye tracking function enabled as the tracking cameras may also employ infrared light,^94^ posing potential interference with fNIRS measurements.

On the other hand, CAVE iVR is often considered to be better in creating highly immersed experiences as it offers a wider viewing angle, higher screen resolution, and more freedom of user movement (which may, however, introduce more motion artifacts in the fNIRS data at the same time).^90^ CAVE iVR provides a more natural sense of embodiment as users can see their own bodies during the iVR experience.^31^^,^^95^ Its spacious environment can accommodate additional pieces of equipment, such as a driving simulator or a flight cockpit simulator, to offer realistic visual and haptic feedback. Unlike HMDs, which are generally intended for a single user, CAVE VR can simultaneously immerse multiple individuals in the same environment,^92^ facilitating fNIRS research that aims at exploring brain coupling among users, as seen in hyperscan setups.^24^ CAVE iVR also has fewer issues with regards to the competition for space over the user’s head. As seen from previous iVR-fNIRS publications, CAVE iVR permitted higher numbers of installed fNIRS channels (an average of 46 versus 22 with an HMD) and larger sampling areas.

Comparison studies reported mixed results over user behavior and task performance during iVR using HMD or CAVE systems. CAVE iVR was favored in a few early studies because of higher reported levels of presence and stronger emotional responses in subjects,^96^^,^^97^ whereas others found that participants rated HMD higher in terms of presence and showed better task performance.^98^^,^^99^ Some studies suggested a minimal difference between the two systems regarding user attention, engagement, and comfort.^95^^,^^100^^,^^101^ These inconsistencies might be partly attributed to the continuous evolution of iVR technologies, particularly the HMDs, which can dramatically impact user experiences. Only one study has directly compared HMD and CAVE setups in the context of fNIRS applications; however, no definitive conclusion was drawn.^31^

### 5.3 Limitations in Current iVR-fNIRS Studies and Future Work

#### 5.3.1 Study design

Despite the growing number of iVR-fNIRS publications, there remains a notable absence of large-scale, extensive investigations within the existing literature that delineate brain functions during iVR experiences, especially studies making comparisons between immersive environments and non-immersive setting such as conventional computer screens. Indeed, previous studies have shown that the human brain may respond differently to stimulation presented in two-dimensional versus three-dimensional environments.^102^^,^^103^ Large-scale comparison studies in the future will be crucial for demonstrating the advantages of using iVR to elicit brain responses in fNIRS research. It might also be beneficial to achieve larger brain coverage and implement more comprehensive analytical procedures in iVR-fNIRS, as current studies primarily focus on sampling from the prefrontal regions and rely on offline, direct assessment of HbO and/or HbR concentrations.

Another notable constraint in current iVR-fNIRS literature is the lack of standardized task designs and experimental procedures. This may limit the ecological validity of iVR-fNIRS, mirroring the difficulty faced in real-world neuroscience investigations.^104^ For instance, brain assessments using imaging techniques usually require the repetition of stimuli/tasks using block or event-related designs to achieve a sufficient signal-to-noise ratio.^105^ Moreover, it is common in iVR studies for participants to undergo pre-training or familiarization with the virtual environment and operations prior to the actual experiment to ensure safe and precise delivery of the iVR experience. This may pose challenges for iVR tests that are designed to reflect real-world environments, such as those evaluating users’ social behaviors and psychological effects. The repetitive stimulations may lead to responses that differ from those in a real-world, unrestrained condition (e.g., due to habituation effect^106^). In our review, we observed that many iVR-fNIRS studies of this type employed a single continuous task paradigm or a limited number of stimulation blocks/sessions incorporating varying levels of iVR environmental change across sessions^35^^,^^50^^,^^60^ (Tables 1 and 2). Those investigations often included one or several control conditions, and the analysis of results relied more on parametric models to assess the brain signal contrast, such as GLM-based beta-values/t-values and channel-wise functional connectivity coefficients. Future work is needed to delineate the impact of condition and task designs in iVR studies.

#### 5.3.2 Physiological interferences and motion artifacts

The inherent nature of light propagation in fNIRS measurements introduces physiological interferences from extracerebral layers, including signals associated with heartbeats, respiration, and blood pressure variations.^107^^,^^108^ In the context of iVR-fNIRS setups, these interferences become more pronounced due to the diverse iVR visual stimulations and interactions involving users in standing or walking conditions.^109^ Movements such as head rotation, arm-raising, use of iVR controllers, and other body displacement not only induce increased motion artifacts and confounding neurological processes but also amplify interfering components arising from heightened body physiological responses in the measured fNIRS signals.^105^ Addressing these challenges is crucial in future iVR-fNIRS investigations to ensure the reliability of findings and prevent false discoveries.^110^ Strategies employed in the reviewed studies included adopting lower low-pass temporal filtering cutoff frequencies (e.g., 0.1 or 0.2 Hz)^28^^,^^32^^,^^42^^,^^44^^–^^47^^,^^51^^,^^53^^,^^55^^,^^60^^,^^61^ in contrast to the recommended 0.5Hz,^111^ various motion correction methods,^35^^,^^38^^,^^39^^,^^44^^,^^55^^,^^56^^,^^62^ principal component analysis for signal component separation,^57^^,^^61^ and pre-whitening and least-square regression-based approaches to eliminate intrinsic signal auto-correlations.^33^^,^^54^^,^^58^^,^^59^ Some studies incorporated additional measures to account for systemic physiological effects, such as the inclusion of short-separation fNIRS channels.^49^^,^^54^ However, this practice was not common, potentially due to constraints related to limited head space and the complexity of system setups necessitated by the integration of both fNIRS and iVR hardware. Efforts to refine and standardize methodologies in handling physiological interferences and motion artifacts will be essential for advancing the robustness of iVR-fNIRS investigations.

#### 5.3.3 Realistic interactions – parallels in real life

Although iVR provides a rich environment for user interactions, the majority of current iVR-fNIRS studies, including those with CAVE iVR, limited the level of interaction of their participants to predefined environmental objects or computer-controlled avatars that do not adapt to user inputs. Enhancing communication in iVR among multiple participants or between participants and researchers could be advantageous in the study of social interactions, reinforcement/feedback mechanisms, adaptive behavior, and various other psychological effects such as those in the novel avatar therapy, in which a conversation between the therapist and the patient needs to be established.^112^

#### 5.3.4 iVR side effects

Motion sickness and cybersickness are substantial challenges in iVR-fNIRS studies. The complex multisensory stimulations in iVR can disrupt users’ perception of their position, orientation, and locomotion, resulting in sensory conflicts from visual inputs and the vestibular system.^113^ Studies have indicated that ∼60% to 95% of iVR users may experience varying degrees of sickness symptoms,^114^ such as nausea, dizziness, headache, and sweating, regardless of whether an HMD or CAVE system was used.^115^^,^^116^ Depending on the number of turns in a navigational VR environment, people may feel mild to significant degrees of motion sickness; for example, in a study comparing older and younger adults’ spatial orientation, about 10% of the participant in each age group could not complete the experiment either using HMD or a laptop display due to motion sickness, and another 10% of the remaining participants could not continue using HMD but finished the experiments using the laptop screen.^117^ These symptoms can have adverse effects in iVR-based brain studies, including disruptions in user brain functions, alterations in behavior and task performance, reduced immersion levels, and a notable rate of participant withdrawal. To address these effects, several iVR-fNIRS studies have implemented inclusion/exclusion criteria to select participants who do not experience motion-related sickness in iVR.^45^^,^^58^ Other studies opted to discard datasets that might be affected by motion sickness^43^ or limited the total exposure time of participants to iVR.^50^^,^^62^ Nevertheless, these mitigating measures introduce additional constraints on study execution and generalizability of results. Future research may explore strategies to control factors in an iVR environment design that lead to user motion sickness/cybersickness, such as increasing head stability,^118^ minimizing user rotation and acceleration rates, implementing dynamic field of view adjustments during virtual movement,^119^ and reducing display latency and flickering.^69^ Studies have also demonstrated that enhancing user controllability during iVR experiences can significantly alleviate motion sickness symptoms. Notably, iVR locomotive controllers (such as an environmental navigation chair that translate the movement of a wheelchair) have been shown to be able to reduce user sickness by providing vestibular and proprioceptive sensory inputs that match the iVR stimuli while ensuring movement accuracy.^113^^,^^120^

#### 5.3.5 Other technical issues

There may be a number of additional technical concerns in today’s iVR-fNIRS setup. First, in studies employing HMDs, the headset and its connection cables are mounted on top of the necessary fNIRS components (e.g., cap, optodes, holders, and optical fibers), resulting in an extra 0.5 to 1 kg of weight loaded on the subject’s head. The strap used to stabilize the VR headset can increase the pressure of the fNIRS cap and optode holders, potentially causing user discomfort or even pain after long-term use.^7^ Moreover, the multisensory inputs and high level of immersion associated with HMD or CAVE iVR can be demanding,^65^ which, combined with the added equipment weight and pressure on the head, can further enhance visual fatigue, muscular fatigue, acute stress, cybersickness, and mental overload among users.^121^ Several intrinsic limitations of fNIRS technology may also have impacts in iVR-fNIRS studies. For instance, iVR has been reported to be useful in human emotion studies as it is able to intensify user emotional response with realistic environments and stimulations.^122^ However, fNIRS measurements are restricted to the superficial cortex, limiting our ability in studying deep brain structure functions involved in emotion processing.^37^^,^^44^ The installation of headsets in HMD iVR may complicate fNIRS data calibration and acquisition, making the acquired signals more susceptible to contamination from hair and sensitive to user movement.

## 6 Conclusion

The combination of the two emerging techniques, iVR and fNIRS, holds immense promise in neuroscience research and therapy. iVR stands out as a low-cost yet potent tool, enabling researchers to deliver precisely controlled multisensory stimuli that closely mimic real-world scenarios, enhancing the ecological validity of subjects’ responses and behaviors. On the other hand, fNIRS establishes real-time brain assessment concurrently with iVR stimulations, while offering flexibility to adapt to iVR requirements across diverse experimental and clinical contexts. Future advancement of iVR-fNIRS, including the development of lightweight and compact wearable units, more comprehensive online data processing methodologies, real-time communication capabilities, motion sickness/cybersickness reduction techniques, and large-scale comparative studies will likely unlock its potential across various domains, encompassing VR-based neurofeedback systems, advanced brain-computer interfaces, hyperscan research, and the more recent “metaverse” development. In this rapidly evolving field of brain research, in which multidimensional stimulation and robust brain evaluations are imperative for progress, iVR-fNIRS may emerge as a useful tool to offer valuable insights that can advance our understanding of the human mind and its capabilities.

## Data Availability

Data sharing is not applicable to this article as no new data were created or analyzed in this study.
